# *Salicornia ramosissima* Biomass as a Partial Replacement of Wheat Meal in Diets for Juvenile European Seabass (*Dicentrarchus labrax*)

**DOI:** 10.3390/ani14040614

**Published:** 2024-02-14

**Authors:** André Barreto, Ana Couto, Daniel Jerónimo, Adriana Laranjeira, Bruna Silva, Catarina Nunes, Ana C. S. Veríssimo, Diana C. G. A. Pinto, Jorge Dias, Mário Pacheco, Benjamin Costas, Rui J. M. Rocha

**Affiliations:** 1 Riasearch Lda, Cais da Ribeira de Pardelhas, no. 21, 3870-168 Murtosa, Portugal; danieljeronimo@riasearch.pt (D.J.); adrianalaranjeira@riasearch.pt (A.L.); brunasilva@riasearch.pt (B.S.); ruirocha@riasearch.pt (R.J.M.R.); 2 Centro Interdisciplinar de Investigação Marinha e Ambiental (CIIMAR), Terminal de Cruzeiros do Porto de Leixões, Av. General Norton de Matos s/n, 4450-208 Matosinhos, Portugal; acouto@fc.up.pt (A.C.); catarinafcupb@gmail.com (C.N.); bcostas@ciimar.up.pt (B.C.); 3 LAQV-REQUIMTE, Departamento de Química, Campus Universitário de Santiago, Universidade de Aveiro, 3810-193 Aveiro, Portugal; carolinaana@ua.pt (A.C.S.V.); diana@ua.pt (D.C.G.A.P.); 4 Sparos Lda, Área Empresarial de Marim, Lote C, 8700-221 Olhão, Portugal; jorgedias@sparos.pt; 5 CESAM, Departamento de Biologia, Campus Universitário de Santiago, Universidade de Aveiro, 3810-193 Aveiro, Portugal; mpacheco@ua.pt; 6 Instituto de Ciências Biomédicas Abel Salazar (ICBAS-UP), Universidade do Porto, 4050-313 Porto, Portugal

**Keywords:** *Salicornia ramosissima*, residues, by-products, circular economy, European seabass, aquafeeds

## Abstract

**Simple Summary:**

The production of halophyte plants, like *Salicornia ramosissima*, is increasing as they are highly valued for human consumption and due to their ability to grow in unutilized cultivation areas, like saline soils. However, only the green tips are commercialized, while the remaining plant is considered a residue. This study aimed to explore the potential of these salicornia by-products to be included in aquafeeds for juvenile European seabass, partially replacing wheat meal. The results obtained indicate that this substitution is possible with no apparent adverse effects on the fish and on the economic viability of the feeds. Additionally, the inclusion of salicornia in the diets was related to the decrease in compounds in fish muscle usually used as stress biomarkers and with the increase in DHA levels, one of the most relevant Omega-3 fatty acids for human nutrition. Therefore, data from this study suggest that salicornia by-products are a viable alternative to partially replace wheat meal in diets for juvenile European seabass, allowing salicornia farmers to valorize a residue and to implement the principles of circular economy in halophyte farming and the aquaculture industry.

**Abstract:**

The green tips of *Salicornia ramosissima* are used for human consumption, while, in a production scenario, the rest of the plant is considered a residue. This study evaluated the potential of incorporating salicornia by-products in diets for juvenile European seabass, partially replacing wheat meal, aspiring to contribute to their valorization. A standard diet and three experimental diets including salicornia in 2.5%, 5% and 10% inclusion levels were tested in triplicate. After 62 days of feeding, no significant differences between treatments were observed in fish growth performances, feeding efficiency and economic conversation ratio. Nutrient digestibility of the experimental diets was unaffected by the inclusion of salicornia when compared to a standard diet. Additionally, salicornia had significant modulatory effects on the fish muscle biochemical profiles, namely by significantly decreasing lactic acid and increasing succinic acid levels, which can potentially signal health-promoting effects for the fish. Increases in DHA levels in fish fed a diet containing 10% salicornia were also shown. Therefore, the results suggest that salicornia by-products are a viable alternative to partially replace wheat meal in diets for juvenile European seabass, contributing to the valorization of a residue and the implementation of a circular economy paradigm in halophyte farming and aquaculture.

## 1. Introduction

The halophyte plant *Salicornia ramosissima*, popularly known as green samphire, is widely distributed in the salt marshes of the Mediterranean region. It is an annual species that grows at low salinities, although it can tolerate high salt concentrations in the soil. From a production perspective, it can be irrigated with seawater, allowing the utilization of unexploited cultivation areas, which is an advantage over most cultivated plants that can be exploited [1,2,3]. Salicornia is considered a gourmet product for human consumption, with its fresh branch tips being highly appreciated [3,4,5,6,7]. The nutritional profile of *S. ramosissima* is characterized by considerable amounts of protein (5–10% DW), *n*-3 and *n*-6 polyunsaturated fatty acids (mainly α-linolenic and linoleic acid) and minerals (such as sodium, potassium, calcium, magnesium, iron and manganese) [4,5]. Their richness in bioactive secondary metabolites, which can have health-promoting effects for the consumer, is a distinctive quality of these plants, exhibiting a significant antioxidant and anti-inflammatory potential due to their total phenolic content [5,8]. In addition, seeds of Salicornia spp. contain considerable levels of lipids (≈26–33%) and proteins (≈31%) [9].

While the tenderest stems of salicornia are intended for human consumption, there are currently no commercial applications for the remaining parts of the plant, often considered a residue. However, this salicornia biomass has enormous potential to be used for animal nutrition, potentially as a feedstock ingredient that would reduce the use of edible cereal crops like wheat. The production of this cereal in 2021 reached 776 million tons, with around 70% being directly used for human nutrition, around 20% for livestock feed and around 10% for seeds, industrial applications and other uses [10]. While wheat consumption is expected to expand globally, albeit at a below-average pace, a decrease in feed applications is anticipated to cause a 0.4% decline in total wheat utilization in 2022/2023, predominantly driven by the escalation of market prices [10]. In the current scenario, where a progressive increase in human population numbers is foreseen, the development of alternative food and feed products by exploiting resources not usually used by traditional production (e.g., halophyte plants in saline soils, saline irrigation) is imperative. Furthermore, the valorization of residues such as these salicornia by-products would contribute to implementing circular economy principles in halophyte farming and the aquaculture industry, increasing the sustainability of both industries.

The European seabass (*Dicentrarchus labrax*) is one of the most representative species in the Mediterranean aquaculture industry, with its global production reaching almost 300 thousand tonnes (USD 1.8 million in value) in 2021 [11,12]. The intensive production of this carnivorous species relies on high protein diets whose formulation has traditionally depended on fish meal (FM) as the major ingredient [13,14,15]. Nevertheless, FM preponderance in aquafeeds has been progressively reduced due to limited market availability and sustainability issues [16,17]. The European seabass has a high tolerance to vegetal ingredients and high replacement levels of FM in formulated diets have been successfully achieved with no adverse effects on fish growth performances, as thoroughly reviewed by Oliva-Teles et al. [18]. Although salicornia protein contents are too low to replace FM directly, its by-products may serve as a source of carbohydrates in aquafeed formulations, while potentially conferring functional properties to the feed due to their richness in bioactive compounds.

In this context, the present study aimed to evaluate the potential of including *S. ramosissima* aerial by-products in aquafeeds as a partial replacement of wheat meal in diets for juvenile European seabass. For this purpose, fish growth performance, survival, body composition, muscle biochemical profiles, nutrient digestibility and the economic viability of feeds were assessed.

## 2. Materials and Methods

### 2.1. Dietary Treatments

Four experimental diets were evaluated in triplicates. A control diet (CTRL) was formulated to meet the nutritional requirements of juvenile European seabass, containing 35% fish meal, 16.3% wheat meal, 13% soy protein concentrate and 10% wheat gluten as the main protein sources and 5.7% rapeseed oil and 5.2% fish oil as the main lipid sources. Additionally, three experimental diets based on CTRL were used, differing only in the ingredient formulation by replacing wheat meal with salicornia whole plant biomass at 2.5% (SAL2.5), 5% (SAL5) and 10% (SAL10) inclusion levels, respectively. Salicornia biomass was obtained from Praia da Areia Branca, Torreira, Portugal (40°46′22.1″ N 8°39′29.5″ W), in June 2020. The roots and green tips, which are used for human consumption, were separated and not used in the current trial, so the biomass included in the experimental diets would resemble the by-products of salicornia production. The remaining parts of the plant were then dried in mesh mats that allowed air circulation, to prevent any degradation promoted by humidity. Wheat gluten levels were slightly adjusted in the experimental diets to ensure that protein levels remained similar between diets. The experimental diets’ formulation, production cost and nutrient composition analysis can be seen in Table 1 and Table 2, respectively.

Diets were manufactured by extrusion at Sparos Lda (Olhão, Portugal) facilities. All powder ingredients were mixed according to the target formulation in a double-helix mixer (model 500L, TGC Extrusion, Roullet-Saint-Estèphe, France) and ground (below 400 µm) in a micropulveriser hammer mill (model SH1, Hosokawa-Alpine, Augsburg, Germany). Diets (pellet size: 1.2 and 2.0 mm) were manufactured with a twin-screw extruder (model BC45, Clextral, Firminy, France) with a screw diameter of 55 mm. Extrusion conditions: feeder rate (80 kg/h), screw speed (255 rpm), water addition in barrel 1 (340 mL min^−1^), temperature barrel 1 (36 °C) and temperature barrel 3 (110–112 °C). Extruded pellets were dried in a vibrating fluid bed dryer (model DR100, TGC Extrusion, Roullet-Saint-Estèphe, France). After cooling, oils were added by vacuum coating (model PG-10VCLAB, Dinnissen, Sevenum, The Netherlands). Coating conditions were pressure of 700 mbar, with spraying time under vacuum of approximately 90 s and a return to atmospheric pressure at 120 s. After coating, diets were packed in sealed plastic buckets and stored at room temperature.

### 2.2. Fish Rearing and Sampling

European seabass juveniles (7.3 ± 0.1 g) originating from Sonrionansa (Cantábria, Spain) were transported to Riasearch Lda facilities (Murtosa, Portugal) by a legally authorized ground carrier in live fish transportation. These were distributed to 12 tanks with 200 L part of a state-of-the-art 40 m^3^ clear water recirculating aquaculture system (RAS). Each tank was stocked with 80 individuals. Water parameters were measured daily using commercial equipment. During the experimental period, temperature was maintained at 21.6 ± 0.2 °C, dissolved oxygen at 6.4 ± 0.6 mg L^−1^, salinity at 18.2 ± 0.2 g L^−1^, pH at 7.5 ± 0.2, NH_3_ at 0.0 ± 0.0 mg L^−1^ and NO_2_ at 0.1 ± 0.1 mg L^−1^.

Fish were kept under a 14 h light:10 h dark photoperiod and fed by hand until visual satiety with 3 daily meals for 62 days. Feed given to each tank was quantified daily. The feed size was 1.2 mm for the first 30 days and 2 mm for the remaining feeding period.

At the end of the experiment (Day 62), fish from each tank were weighed to determine mean body weight, specific growth rate (SGR), feed intake, feed conversion ratio (FCR), economic conversion ratio (ECR, EUR spent in feed per Kg of biomass gain, for three different hypothetical salicornia by-products market price scenarios: 0%, 50% and 80% of wheat meal cost) and survival. Furthermore, 10 fish per tank were euthanized with a lethal dosage of anesthetic: 5 were sampled for whole-body composition analysis, and muscle samples were taken from 5 additional fish for biochemical profiling. Samples for whole-body composition and biochemical profiling were kept at −20 °C and −80 °C until further analysis, respectively. Fish were fasted overnight prior to any sampling procedures.

#### Digestibility Trial

In parallel, a digestibility trial was conducted to collect feces for an analysis of nutrient apparent digestibility. The trial was performed in a clear water RAS, equipped with 12 fiberglass tanks of 70 L water capacity designed according to the Guelph system (each tank provided in the outlet with a feces settling column). Sixteen fish averaging 60.2 ± 0.8 g were allocated to each tank and diets were randomly assigned to triplicate groups of these fish. Fish were fed by hand twice daily until apparent visual satiation. Fish were adapted to the rearing conditions and diets for 5 days. Thereafter, feces were collected daily until enough feces had been gathered to perform the required analysis, and then pooled for each tank and frozen at −20 °C until analysis. Water parameters were controlled daily using commercial equipment and kept similarly to those described for the growth trial.

### 2.3. Bromatological Analyses

Bromatological analyses of the experimental diets, fish carcasses and feces were performed following the Association of Official Analytical Chemists procedures [19]. Briefly, dry matter was determined by drying samples at 105 °C in an oven until constant weight; ash by incineration at 450 °C for 16 h in a muffle furnace; crude protein content (N × 6.25) by the Kjeldahl method after acid digestion using a Kjeltec digestion and distillation unit (model 1015 and 1026, Tecator Systems, Höganäs, Sweden, respectively); lipid content by petroleum ether extraction (Soxtec HT System, GeminiBV, Apeldoorn, The Netherlands); and gross energy, by direct combustion in an adiabatic bomb calorimeter (PARR model 1261, PARR Instruments, Moline, IL, USA). Yttrium oxide was determined as follows: sample digestion with HNO_3_ + H_2_O_2_ + HF in a microwave, dilution and analysis on an ICP-SFMS (Element^TM^ 2 ICP-SFMS, ThermoFisher Scientific^TM^, Carlsbad, CA, USA). Feces were analyzed for protein, lipids, energy and yttrium oxide (Y_2_O_3_) contents. Apparent digestibility coefficients (ADC) were calculated according to the following formula: ADCnutrient = 100 − [100 × (Y_2_O_3_ diet/Y_2_O_3_ feces) × (nutrient feces/nutrient diet)].

### 2.4. Muscle Biochemical Profile

Muscle samples were cut into small pieces and kept in an oven at 35 °C to dehydrate. When the weight of the samples stabilized, they were macerated using a mill. Samples were then derivatized in triplicate by adding 250 μL of pyridine, 250 μL of BSTFA (N,O-bis(trimethylsilyl)trifluoroacetamide), 50 μL of trimethylsilyl chloride, 200 μL of 1 mg/mL of triacontane (internal standard) and 250 μL of dichloromethane to 15 mg of biomass. The mixtures were maintained at 70 °C for 30 min and then transferred to vials. Samples’ derivates were injected in duplicates into a GC-MS (Gas Chromatography–Mass Spectrometry) apparatus.

GC–MS analysis of each silylated sample was performed using a GC–MS QP2010 Ultra Shimadzu (University of Aveiro, Aveiro, Portugal) equipped with a ZB-5ms J & W capillary column (30 m × 0.25 mm × 0.25 μm). Samples were injected with a split ratio of 1:10 and helium as carrier gas with a flux of 1.19 mL/min. The injector temperature was 320 °C and the transfer-line temperature was 200 °C. The temperature of the column was maintained at 70 °C for 5 min and then increased, first at 4 °C/min until 250 °C, followed by an increase of 2 °C/min until 300 °C, where it was maintained for 5 min. The mass spectrometer was operated in the electronic impact (EI) mode with an energy of 0.1 kV, and data were collected at a rate of 1 scan/s over a range of *m*/*z* 50–1000. The performed chromatography lasted 80 min and the standards were analyzed separately by GC–MS under the same chromatographic conditions. The compounds were identified based on a direct comparison with the mass spectra database libraries (NIST14 Mass Spectra and WILEY Registry TM of Mass Spectra Data), and when possible pure standards of each compound were subjected to the same derivatization procedure and injected in the same chromatographic conditions. The quantification of each metabolite present in each replicate was obtained from calibration curves acquired by injection of solutions of known concentration of each standard. Standard concentrations were chosen to ensure the quantification of each compound in the samples through the interpolation of the calibration curve.

### 2.5. Data Analysis

Specific growth rate (SGR,% weight day^−1^) was calculated as SGR = (e^g^ − 1) × 100, where g = (lnWf − lnWi) × t^–1^. Wf and Wi correspond to the final and initial weights (g), respectively, and t corresponds to time in days. Feed conversion ratio (FCR) was calculated as FCR = (Fi/Wg), where Fi corresponds to feed ingested (g) and Wg to the mean weight gain (g). Feed intake (% average body weight (ABW) day^−1^) was calculated as feed intake = (Fi/((Wi + Wf)/2)/t) × 100, where Fi corresponds to feed ingested (g), Wi and Wf correspond to the initial and final weights (g), respectively, and t corresponds to time in days. The economic conversion ratio (ECR) was calculated as ECR = ((Fi × C)/Wg), where Fi corresponds to feed intake (Kg), C to diet price (EUR Kg^−1^) and Wg to the mean weight gain (Kg). Survival was expressed as a percentage and calculated as S = (Sf/Si) × 100, where Si and Sf correspond to the initial and final number of individuals in the tanks, respectively.

Differences in growth performance, feeding efficiency, survival, ECR, whole body composition, digestibility and muscle biochemical profiles between dietary treatments were evaluated using one-way ANOVA, followed by Tukey’s HSD multiple comparison tests. Kruskal–Wallis one-way analysis of variance tests, followed by Wilcoxon pairwise comparison tests, were used when data did not comply with the one-way ANOVA’s assumptions. Results were expressed as means ± standard deviation (SD). In results expressed as percentages, an arcsine transformation was performed prior to any statistical test: T = ASIN (SQRT (value/100)). The significance level considered was *p* < 0.05 for all tests performed.

## 3. Results

### 3.1. Zootechnical Parameters

At the end of the experiment (Day 62), no significant differences between treatments were found in fish final mean body weight, SGR, feed intake, FCR and survival values (Table 3).

### 3.2. Economic Conversion Ratio

As for ECR, no significant differences between treatments were found in any of the three hypothetical scenarios for salicornia market price (Table 4).

### 3.3. Whole-Body Composition

Moreover, significant differences between treatments were found in the fish whole-body composition. Fish fed the SAL2.5 and SAL5 diets had significantly higher energy content than those fed CTRL (Table 5).

### 3.4. Digestibility

As for nutrient digestibility, the ADC of dry matter was significantly higher in diets SAL2.5 and SAL5 than in CTRL and SAL10, while the ADC of protein was significantly higher in SAL2.5 than in SAL10 (Table 6).

### 3.5. Muscle Biochemical Profiles

Regarding the muscle biochemical profiles, the inclusion of salicornia biomass in the experimental diets had profound modulatory effects on most compounds analyzed, with only 11,14-Eicosadienoic acid and arachidonic acid levels being similar in all dietary treatments (Table 7).

## 4. Discussion

This study aimed to evaluate the potential of incorporating *S. ramosissima* aerial by-products in diets for juvenile European seabass, partially replacing wheat meal, aspiring to contribute to the valorization of these residues and to implement principles of circular economy in halophyte farming and aquaculture. The European seabass was chosen as the model organism for this study as it is one of the most produced finfish species in Mediterranean aquaculture and, despite being a carnivorous species, has considerable tolerance to feeds formulated with high proportions of vegetal ingredients, as long as their indispensable amino acid profile is appropriate [18,20]. Overall, the survival, growth performance and feeding efficiency results obtained in the current study can be considered very satisfactory and indicate that proper zootechnical conditions were maintained during the experimental trial and that the feeds utilized were adequate for the developmental stage of the fish. The results obtained were superior to those reported by Peres and Oliva-Teles [21], who studied the utilization of dietary protein by European seabass juveniles (5.5 g) at 18 °C and 25 °C, describing final weights of up to 29.9 g after 12 weeks; Moreira et al. [22], who described an increase in fish weight from 15 g to up to 45 g in 10 weeks, at 25 °C, utilizing different carbohydrate levels in aquafeeds; Gasco et al. [23], who tested the inclusion of *Tenebrio molitor* meal in diets for European seabass juveniles (5.2 g) for 70 days, at 19.5 °C, with fish achieving final weights up to 22.1 g with the control diet; Coutinho et al. [24], who tested the effects of dietary methionine and taurine supplementation in the diets of European seabass juveniles (6.9 g) at 23 °C, describing final weights of up to 28.4 g after 85 days; and Torrecillas et al. [25], who reported an increase in fish weight from around 13 g to up to 44.4 g in 70 days, at 23 °C, utilizing feeds supplemented with an arachidonic acid rich oil.

The inclusion of salicornia in the diets, in proportions up to 10%, did not compromise their adequacy, as fish survival, growth performance and feeding efficiency values were identical to those obtained when using the control diet. Additionally, no negative effects were observed in the fish whole body composition and digestibility of nutrients, indicating that the replacement of wheat meal with salicornia did not affect the experimental diets’ bioavailability of nutrients and their metabolic utilization by the fish. In fact, when 2.5% and 5% of *S. ramosissima* biomass were used in feeds, an increase in fish whole body energy levels was verified in relation to those fed the standard diet, which can probably be explained by significantly higher levels of ADC of dry matter and a tendency for higher levels of protein ADC in the SAL2.5 and SAL5 dietary treatments.

Furthermore, the analysis performed on the muscle of the fish revealed that the inclusion of salicornia biomass in the experimental diets had significant modulatory effects on the levels of the alcohols, amino acids, organic acids, saturated fatty acids, unsaturated fatty acids and sterols that were evaluated. However, these results are complex and a pattern of the relationship between the variations observed and the inclusion of salicornia in the diets is difficult to establish. The alterations in the levels of most compounds analyzed did not affect fish zootechnical parameters, but further studies that delve into what these represent for the fish health status and the nutritional benefits for the consumer are still necessary. Still, it should be noted that lactic acid levels in the fish muscle significantly decreased as the proportions of salicornia in the diets increased to 5% and 10%. Lactic acid has been used to measure stress levels in fish and increases have been associated with handling stress in striped bass *Morone saxatilis* juveniles [26]; with hypoxia stress in channel catfish *Ictalurus punctatus* [27] and in largemouth bass *Micropterus salmoides* [28]; and with air exposure and physical exhaustion in red throat emperor *Lethrinus miniatus* [29]. Therefore, these results suggest that salicornia may have contributed to an increase in the fish resilience to stress, resulting in a decrease in lactic acid production, as these were subject to sampling procedures that included netting, brief air exposure and handling prior to being euthanized with a lethal dosage of anesthetic, and muscle samples were taken. Concomitantly, succinic acid levels in fish muscle tissue increased linearly with salicornia proportions, which again may indicate a health-promoting effect for the fish through the inclusion of the halophyte in their diets. This organic acid has been used as a dietary additive in aquafeeds and has been linked with the enhancement of growth performances, feeding efficiency, immune activity and/or modulation of the gut microbiota composition of several species of fish and shrimp [30,31,32,33,34]. Additionally, DHA levels were significantly higher in fish fed the diet with the highest salicornia inclusion than in the remaining dietary treatments, while levels of EPA were similar to those of fish fed the standard diet. These results suggest that utilizing aquafeeds containing salicornia by-products would retain, or potentially increase, the benefits associated with the consumption of fish, as DHA and EPA are two of the most relevant Omega-3 fatty acids due to their recognized essential role in human nutrition and development [35,36,37,38,39].

The economic analysis performed revealed that the inclusion of salicornia biomass in the diets, partially replacing wheat meal, did not affect the formulation costs and the ECR values of the experimental diets in the hypothetical scenarios explored. Considering that this salicornia biomass is regarded as a residue with no commercial value and is replacing an edible cereal whose demand and market prices have been increasing in recent years, these outcomes can be deemed extremely promising. Accordingly, these by-products could potentially be marketed at up to 80% of the wheat meal price, making them more appealing for feed manufacturers, and could generate almost EUR 14 million annually for salicornia farmers, representing the valorization of 60 thousand tonnes of residues (considering 10% of inclusion in 600 thousand tonnes of aquafeeds, amounts conservatively estimated by the authors as necessary for European seabass global production). Moreover, salicornia and European seabass production are predominantly established in the Mediterranean area. The proximity of both industries would contribute to the reduction in the environmental footprint of feeds for European seabass, as wheat production in Europe is on a small scale and mostly comes from exports from Asia and the United States of America [40].

## 5. Conclusions

In conclusion, data from this study indicate that *S. ramosissima* biomass can successfully replace wheat meal in diets for juvenile European sea bass for up to 10% of their composition, with no detrimental effects on fish growth performances, feeding efficiency, survival, whole body composition and digestibility coefficients. The inclusion of salicornia in the diets produced intricate modulatory effects on the fish muscle tissue biochemical profiles that require further assessment. Even so, decreases in lactic acid and increases in succinic acid levels were verified, which can potentially signal health-promoting effects for the fish. Increases in DHA levels in fish fed a diet containing 10% salicornia were also shown. Additionally, the results suggest that *S. ramosissima* aerial by-products are an economically viable alternative to wheat meal in aquafeed formulations for European seabass juveniles. This scenario would allow salicornia farmers to valorize a residue that could potentially be marketed at 80% of the wheat meal retail price, while contributing to the implementation of a circular economy paradigm in halophyte farming and the aquaculture industry. Furthermore, the use of these by-products could reduce the environmental footprint of European seabass production due to the proximity of both industries in the Mediterranean region.

## Figures and Tables

**Table 1:** Formulation and production cost of the experimental diets used to culture juvenile European seabass, assuming three different hypothetical market prices for salicornia by-products.

Ingredients (%)	CTRL	SAL2.5	SAL5	SAL10
Fishmeal LT70 ^a^	35.00	35.00	35.00	35.00
Krill meal ^b^	5.00	5.00	5.00	5.00
Soy protein concentrate ^c^	13.00	13.00	13.00	13.00
Wheat gluten ^d^	10.00	10.10	10.10	10.30
Corn gluten meal ^e^	8.00	8.00	8.00	8.00
Wheat meal ^f^	16.30	13.70	11.20	6.00
Vitamin and mineral premix ^g^	1.00	1.00	1.00	1.00
Monocalcium phosphate ^h^	0.78	0.78	0.78	0.78
Yttrium oxide ^i^	0.02	0.02	0.02	0.02
Fish oil ^a^	5.20	5.20	5.20	5.20
Rapeseed oil ^j^	5.70	5.70	5.70	5.70
*Salicornia ramosissima* biomass ^k^	0.00	2.50	5.00	10.00
Production cost (EUR Kg^−1^)				
Salicornia—0.000 EUR kg^−1^	1.164	1.159	1.151	1.140
Salicornia—0.145 EUR kg^−1^	1.164	1.162	1.159	1.155
Salicornia—0.232 EUR kg^−1^	1.164	1.164	1.163	1.163

^a^ Sopropêche, France. ^b^ Aker Biomarine, Norway. ^c^ Soycomil P, ADM, Netherlands. ^d^ VITAL, Roquette, France. ^e^ COPAM, Portugal. ^f^ Molisur, Spain. ^g^ Premix Lda, Portugal. ^h^ Aliphos Monocal, Aliphos, Belgium. ^i^ Amperit, Höganäs Germany GmbH, Germany. ^j^ JC Coimbra, Portugal. ^k^ Riasearch, Portugal.

**Table 2:** Nutrient composition of the experimental diets used to culture juvenile European seabass.

Nutrient Composition (% Dry Matter)	CTRL	SAL2.5	SAL5	SAL10
Dry matter	94.1	96.5	95.5	94.5
Crude protein	52.4	51.4	53.7	52.1
Crude lipids	16.8	16.8	17.3	17.3
Ash	9.0	9.8	10.5	11.3
Energy (KJ g^−1^ DM)	23.4	23.1	23.3	22.7

**Table 3:** Initial and final weight, specific growth rate (SGR), feed conversion ratio (FCR), feed intake and survival of juvenile European seabass fed the experimental diets for 62 days.

	CTRL	SAL2.5	SAL5	SAL10	*p* Value
Initial weight (g)	7.27 ± 0.03	7.24 ± 0.03	7.27 ± 0.09	7.26 ± 0.10	0.959
Final weight (g)	43.70 ± 0.32	43.30 ± 1.28	43.60 ± 0.98	43.50 ± 0.95	0.980
SGR (% day^−1^)	2.93 ± 0.01	2.93 ± 0.05	2.93 ± 0.03	2.93 ± 0.03	0.996
FCR	1.00 ± 0.03	0.99 ± 0.01	0.99 ± 0.02	1.01 ± 0.00	0.470
Feed intake (% ABW day^−1^)	2.32 ± 0.04	2.30 ± 0.02	2.30 ± 0.03	2.36 ± 0.02	0.996
Survival (%)	94.60 ± 4.10	97.10 ± 1.60	97.90 ± 1.20	95.80 ± 1.60	0.856

Results expressed as mean ± standard deviation (*n* = 3 experimental units). For each parameter, one-way ANOVA *p* values are indicated.

**Table 4:** Economic conversion ratio (ECR) of the experimental diets containing salicornia biomass used to culture European seabass juveniles for 62 days.

	CTRL	SAL2.5	SAL5	SAL10	*p* Value
ECR (Salicornia EUR kg^−1^ 0.000)	1.159 ± 0.041	1.147 ± 0.018	1.138 ± 0.023	1.157 ± 0.007	0.727
ECR (Salicornia EUR kg^−1^ 0.145)	1.159 ± 0.041	1.150 ± 0.018	1.146 ± 0.023	1.172 ± 0.007	0.614
ECR (Salicornia EUR Kg^−1^ 0.232)	1.159 ± 0.041	1.152 ± 0.018	1.150 ± 0.023	1.180 ± 0.007	0.483

Results expressed as mean ± standard deviation (*n* = 3 experimental units). ECR was calculated for three hypothetical scenarios, based on different *S. ramosissima* by-product market prices per kg: EUR 0.000, EUR 0.145 (50% of wheat meal cost) and EUR 0.232 (80% of wheat meal cost). For each parameter, one-way ANOVA *p* values are indicated.

**Table 5:** Whole-body composition (% fresh weight) of juvenile European seabass fed the experimental diets containing salicornia for 62 days.

	CTRL	SAL2.5	SAL5	SAL10	*p* Value
Dry matter (%)	29.5 ± 2.5	32.4 ± 1.1	32.4 ± 0.9	30.9 ± 0.7	0.125
Protein (%)	14.5 ± 1.6	16.0 ± 1.3	16.9 ± 1.4	15.7 ± 1.2	0.549
Lipids (%)	12.7 ± 1.2	12.9 ± 0.1	11.8 ± 0.6	12.4 ± 0.7	0.111
Ash (%)	4.5 ± 0.7	5.7 ± 0.7	5.6 ± 0.6	5.4 ± 1.1	0.652
Energy (KJ g^−1^)	7.8 ± 0.8 ^a^	8.9 ± 0.3 ^b^	9.0 ± 0.0 ^b^	8.5 ± 0.2 ^ab^	0.018

Results expressed as mean ± standard deviation (*n* = 3 experimental units). Different superscript letters indicate statistical differences (*p* < 0.05) between treatments in a one-way ANOVA, followed by a Tukey HSD multiple comparison test.

**Table 6:** Apparent digestibility coefficients (ADC) of the experimental diets containing *S. ramosissima*.

	CTRL	SAL2.5	SAL5	SAL10	*p* Value
Dry matter ^1^	81.4 ± 0.5 ^a^	84.2 ± 0.6 ^b^	83.2 ± 0.6 ^b^	80.2 ± 0.8 ^a^	<0.001
Protein ^2^	96.4 ± 0.4 ^ab^	97.1 ± 0.0 ^b^	96.9 ± 0.3 ^ab^	96.3 ± 0.3 ^a^	0.034
Lipids ^2^	98.9 ± 0.5	98.6 ± 0.5	98.6 ± 0.4	98.4 ± 0.6	0.664

Results expressed as mean ± standard deviation (*n* = 3 experimental units). Different superscript letters indicate statistical differences (*p* < 0.05) between treatments in a one-way ANOVA, followed by a Tukey HSD multiple comparison test. ^1^ ADC = 100 − 100 × (% yttrium in feed/% yttrium in feces). ^2^ ADC = 100 − 100 × (% yttrium in feed/% yttrium in feces) × (% nutrient in feces/% nutrient in feed).

**Table 7:** Muscle biochemical profiles of European seabass fed the experimental diets containing *S. ramosissima* for 62 days.

Compounds (μg mg Dry Weight^−1^)		CTRL	SAL2.5	SAL5	SAL10	*p* Value
Alcohols	Glycerol	18.47 ± 0.28 ^bc^	18.76 ± 1.32 ^c^	17.20 ± 0.94 ^b^	13.99 ± 0.44 ^a^	<0.001
Amino acids	Alanine	3.29 ± 0.21 ^b^	2.95 ± 0.08 ^a^	3.02 ± 0.07 ^a^	3.55 ± 0.12 ^c^	<0.001
	Glycine	4.72 ± 0.11 ^b^	3.24 ± 0.36 ^a^	5.13 ± 0.21 ^b^	4.70 ± 0.45 ^b^	<0.001
	Serine	2.95 ± 0.06 ^b^	2.88 ± 0.04 ^a^	2.89 ± 0.02 ^ab^	2.88 ± 0.01 ^a^	0.019
Organic acids	Acetic acid	0.04 ± 0.01 ^a^	0.41 ± 0.02 ^b^	0.35 ± 0.17 ^b^	0.29 ± 0.20 ^b^	0.001
	Lactic acid	23.98 ± 0.50 ^b^	22.38 ± 1.49 ^b^	19.06 ± 1.19 ^a^	18.73 ± 0.34 ^a^	<0.001
	Malic acid	0.39 ± 0.04 ^c^	0.16 ± 0.07 ^a^	0.24 ± 0.03 ^ab^	0.26 ± 0.05 ^b^	<0.001
	Propanoic acid	0.35 ± 0.01 ^d^	0.17 ± 0.01 ^a^	0.22 ± 0.01 ^b^	0.31 ± 0.02 ^c^	<0.001
	Succinic acid	0.22 ± 0.00 ^a^	0.31 ± 0.04 ^b^	0.39 ± 0.01 ^c^	0.74 ± 0.21 ^d^	<0.001
Saturated fatty acids	Heptadecanoic acid	1.06 ± 0.13 ^a^	0.87 ± 0.09 ^a^	0.90 ± 0.03 ^a^	1.31 ± 0.20 ^b^	<0.001
	Myristic acid	9.30 ± 0.38 ^bc^	8.31 ± 0.39 ^a^	8.70 ± 0.16 ^ab^	9.56 ± 0.77 ^c^	0.001
	Palmitic acid	31.84 ± 2.15 ^b^	28.54 ± 3.00 ^ab^	28.59 ± 1.41 ^ab^	26.79 ± 1.02 ^a^	0.003
	Pentadecanoic acid	5.16 ± 0.07 ^ab^	5.09 ± 0.03 ^a^	5.17 ± 0.02 ^ab^	5.23 ± 0.10 ^b^	0.012
	Stearic acid	28.68 ± 3.24 ^b^	24.13 ± 3.84 ^a^	24.65 ± 1.57 ^ab^	23.30 ± 1.43 ^a^	0.013
Unsaturated fatty acids	11,14-Eicosadienoic acid	3.40 ± 0.05	3.36 ± 0.05	3.39 ± 0.03	3.41 ± 0.05	0.273
	11-Eicosenoic acid	5.51 ± 0.48 ^a^	5.03 ± 0.25 ^a^	4.98 ± 0.12 ^a^	6.29 ± 0.37 ^b^	<0.001
	9,12-Octadecadienoic acid	7.68 ± 0.44 ^b^	6.89 ± 0.34 ^a^	6.96 ± 0.13 ^a^	7.61 ± 0.42 ^b^	0.001
	Arachidonic acid	3.40 ± 0.05	3.42 ± 0.04	3.45 ± 0.03	3.40 ± 0.04	0.180
	Docosahexaenoic acid (DHA)	5.77 ± 0.38 ^a^	5.71 ± 0.23 ^a^	5.75 ± 0.17 ^a^	6.35 ± 0.34 ^b^	0.003
	Eicosapentaenoic acid (EPA)	6.92 ± 0.33 ^c^	6.30 ± 0.34 ^ab^	5.84 ± 0.12 ^a^	6.59 ± 0.35 ^bc^	<0.001
	Oleic acid	58.19 ± 2.59 ^b^	56.05 ± 5.17 ^b^	54.59 ± 1.97 ^b^	48.79 ± 2.46 ^a^	0.001
	Palmitelaidic acid	12.76 ± 0.83 ^c^	7.22 ± 0.44 ^a^	7.67 ± 0.25 ^a^	9.09 ± 1.00 ^b^	<0.001
Sterols	Cholesterol	2.66 ± 0.22 ^bc^	2.42 ± 0.26 ^ab^	2.26 ± 0.18 ^a^	2.97 ± 0.24 ^c^	<0.001

Results expressed as mean ± standard deviation (*n* = 3 experimental units). Different superscript letters indicate statistical differences (*p* < 0.05) between treatments in a one-way ANOVA, followed by a Tukey HSD multiple comparison test.

## Data Availability

The data presented in this study are available on request from the corresponding author. The data are not publicly available due to project confidentiality issues.
